# A Comparison of Anteromedial Plating Versus Anterolateral Plating for Humerus Shaft Fractures Using the Anterolateral Approach

**DOI:** 10.7759/cureus.57235

**Published:** 2024-03-30

**Authors:** Vinay Gangwar, Navneet Goel, Apoorv Dua, Vaneet Dhankhar, Mrigank Mathur, Karan Rajpal, Pramod Kumar, Gyanendra Verma

**Affiliations:** 1 Department of Orthopaedic Surgery, Dr. Baba Saheb Ambedkar Medical College and Hospital, New Delhi, IND

**Keywords:** iatrogenic radial nerve injury, diaphyseal shaft humerus fracture, henry's approach, anteromedial plating, anterolateral plating

## Abstract

Background

Plate osteosynthesis is the gold standard treatment for the management of humeral shaft fractures. In the present study, we performed plate osteosynthesis on the anteromedial and anterolateral surfaces using the anterolateral approach to compare the functional outcomes.

Aims and objectives

To study and compare the functional outcome, time to achieve union and associated complications of anteromedial and anterolateral plating in humerus shaft fracture by anterolateral approach.

Methods

This prospective, randomised control study was performed at Dr Baba Saheb Ambedkar Medical College and Hospital, New Delhi, India. This study had 46 patients in total, who were divided into two equal groups at random. All of the fractures in group A were treated using a limited contact dynamic compression plate (LCDCP) on the anterolateral surface using an anterolateral approach, while all of the fractures in group B were corrected using an anteromedial surface using an anterolateral approach using LCDCP. All the patients were followed for six months at regular intervals. At each follow-up, patients were assessed radiologically with X-rays and clinically by Rodriguez-Merchan criteria (RM criteria).

Results and conclusions

The union was achieved in the majority of the cases of the anteromedial plating group within 12 weeks (78.3%) with a mean union time of 11.7±1.5 weeks than the anterolateral group (56.5%) with a mean union time of 12.3±1.8 weeks. Based on functional assessment according to RM criteria, the excellent outcome was achieved in 69.6% and 65.2% of the anterolateral and anteromedial plating groups, respectively. There was no case of non-union and radial nerve palsy in anteromedial plating cases whereas in anterolateral cases one patient did not achieve union and two (8.7%) had radial nerve injury, which recovered completely by the end of the study. An anterolateral approach with anteromedial surface plating on the flat medial aspect of the humerus is a good technique for fixing humeral fractures.

## Introduction

Of all fractures, humeral shaft fractures constitute 1.0% to 5.0% [1]. The humeral shaft extends from the supracondylar ridge distally to the upper border of the pectoralis major muscle's insertion proximally [1]. This fracture has a bimodal distribution, with older women seeing a greater peak and younger patients experiencing peaks more frequently. Plaster immobilization is a conservative treatment option for simple, undisplaced shaft humerus fractures, although it has drawbacks such as angulation and displacement with time. Osteosynthesis or plate fixation is still the gold standard for surgical care [2].

Surgical treatment for humerus shaft fractures has its own advantages. In cases of open fractures, fractures accompanied by neurovascular injury, fractures that do not heal with conservative treatment, patients with polytrauma, multiple fractures, segmental fractures, etc., surgery is taken into consideration. This can be accomplished by the use of an external fixator, intramedullary fixation devices, or plates. Moreover, bridge plating is a treatment option for severely comminuted fractures [3].

Anterolateral or posterior plating is the usual technique for plate osteosynthesis in cases of humeral shaft fracture. In contrast to the medial surface, the lateral side of the humerus is not smooth. Because of the deltoid attachment, the lateral surface has a gentle curvature. As a result, the plate makes poor contact with the bone and needs to be pre-bent.

The radial nerve passes from posterior to anterior at the distal third and middle third junctions of the humerus's lateral intermuscular septum. This may result in damage to the radial nerve due to either bone levers that are accidentally positioned or the plate directly compressing the nerve. The incidence of radial nerve palsy via the posterior approach is 11.0% [1]. Iatrogenic radial nerve injury was noted with an incidence rate of 5.1-17.6%, as radial nerve traverses posterior to the lateral aspect around the distal humerus in anterolateral plating [4]. Many times, a distally fixed plate may be held on the radial nerve causing injury if proper dissection or isolation of the nerve is not done [4].

The anterolateral method suggested by Henry can be used to approach the complete length of the humeral shaft without requiring vision of the radial nerve. When placing the plate on the anterolateral surface, particularly when it is over the middle to the distal third shaft, there is a chance that the nerve may be injured during the retraction of soft tissue or by the implant itself. Restoring the patient's pre-injury level of function and establishing the union with an appropriate humeral alignment are the two main objectives of treating humeral shaft fractures [5].

Because the humerus shaft has both anterolateral and anteromedial surfaces, lateral plating is often referred to as anterolateral plating and medial plating as anteromedial plating. Depending on the fracture site and patient's age, locking compression plates (LCP), dynamic compression plates, and limited contact dynamic compression plates (LCDCP) of sizes 3.5 and 4.5 mm (narrow or broad) can be utilized to treat the fracture [6].

Through an anterolateral approach, stable plate fixation without pre-bending the plate can be accomplished with good union rates and fewer difficulties on the relatively smooth anteromedial surface. When necessary, it offers the advantages of radial nerve exploration [7].

The current study compared the functional results and related problems of plate osteosynthesis performed utilizing the anterolateral technique on the anteromedial and anterolateral surfaces.

## Materials and methods

This is a prospective randomised control study conducted in the Department of Orthopaedic Surgery at Dr Baba Saheb Ambedkar Medical College and Hospital, New Delhi, India. A total of 46 patients were included in the study which was divided into two groups of 23 patients each on the basis of a previously generated random number table. Group A includes patients who underwent osteosynthesis on the anterolateral surface by anterolateral approach and Group B includes patients who underwent osteosynthesis on the anteromedial surface by anterolateral approach. The inclusion criteria were fracture proximal 1/3rd and middle 1/3rd shaft of humerus, age between 16 years and 65 years, unilateral fracture of shaft humerus, closed fracture and Gustilo-Anderson open Grade I fracture, fracture less than four weeks old, and AO type A 1, 2 and 3; B1 and B2 fractures. Exclusion criteria were undisplaced fracture, fracture distal 1/3rd shaft of the humerus, non-union, Gustilo-Anderson open Grade II, IIIa, IIIb, IIIc, AO type B3 and C fractures, patient with any neurovascular deficit, pathological fracture, associated injury in the same limb and previous humeral surgery.

**Figure 1:**
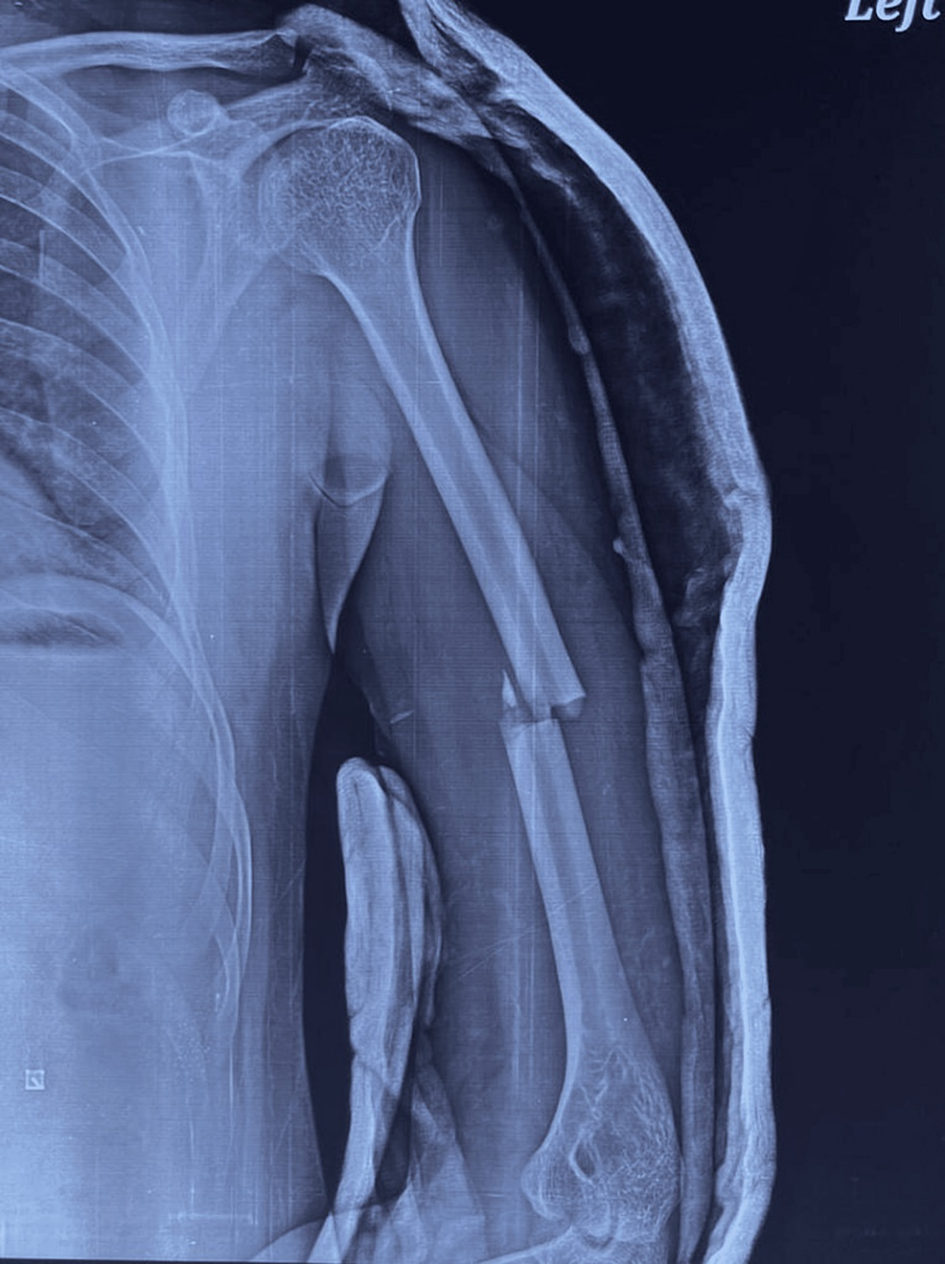
Pre-op radiograph showing fracture shaft of the left humerus.

**Figure 2:**
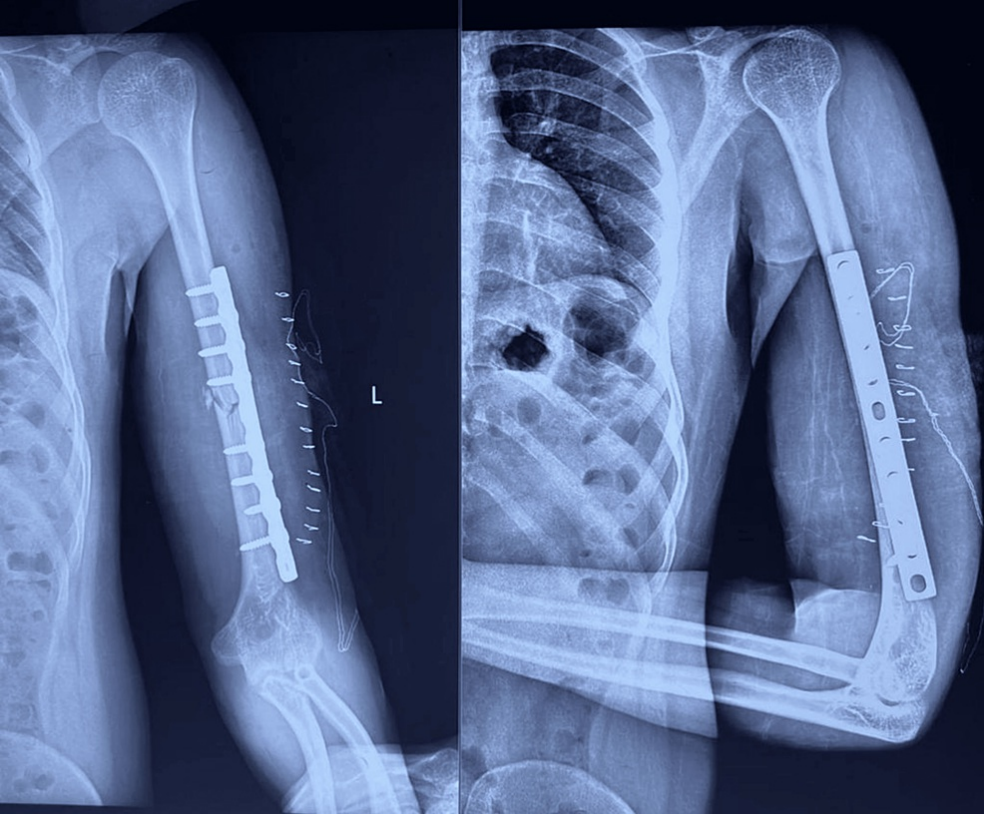
Immediate post-op radiograph showing fixation of the fracture with 4.5mm LCDCP LCDCP: limited contact dynamic compression plates

**Figure 3:**
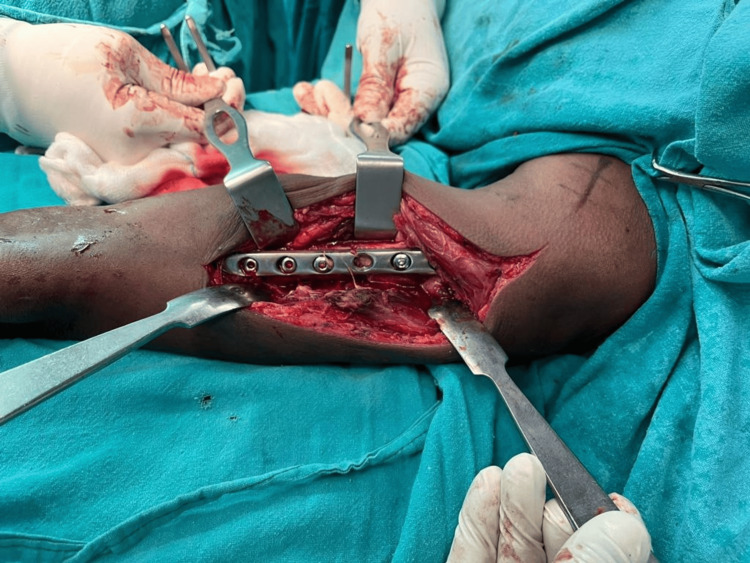
Placement of plate over anterolateral surface

**Figure 4:**
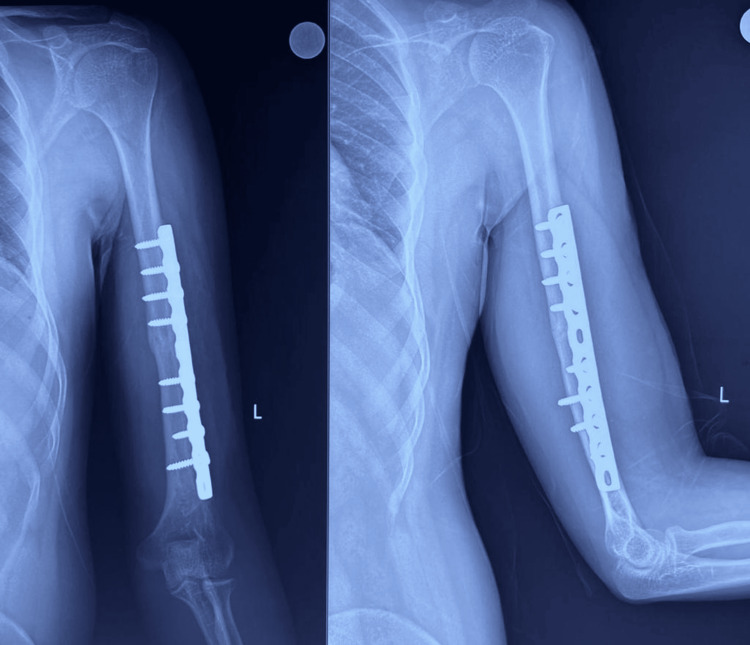
Follow-up radiograph at six months

**Figure 5:**
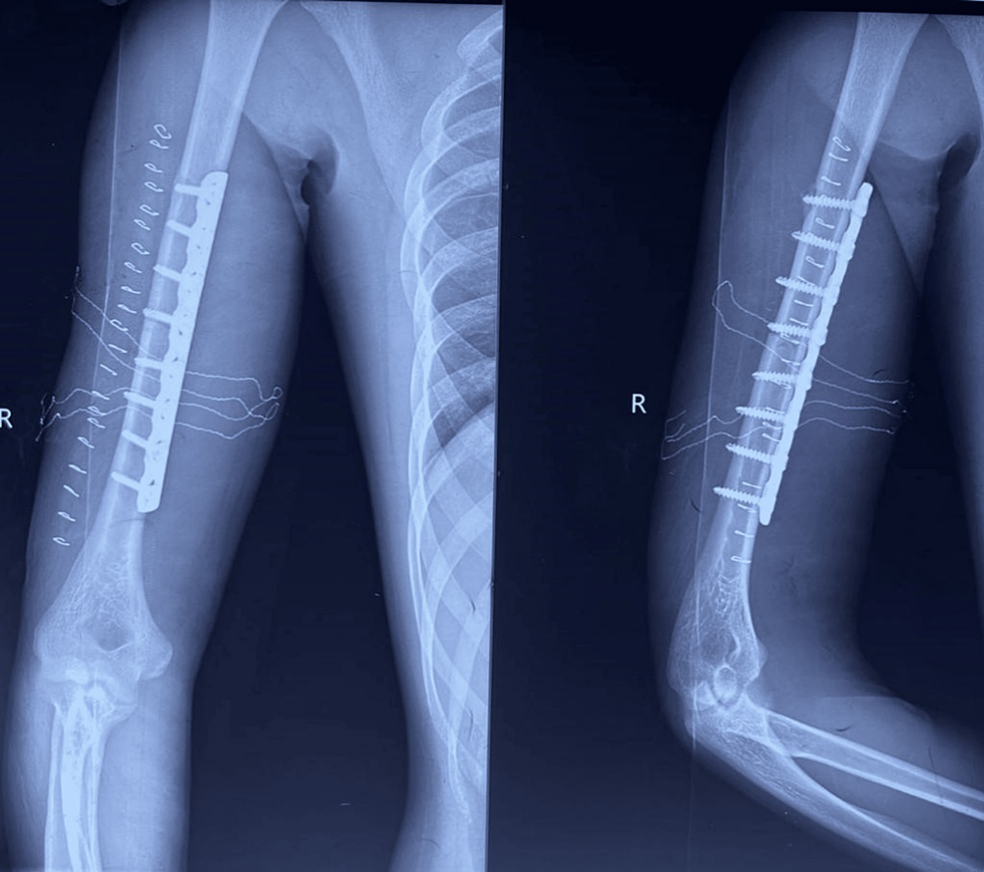
Immediate post-op radiograph showing fixation of humerus shaft fracture with anteromedial plating

**Figure 6:**
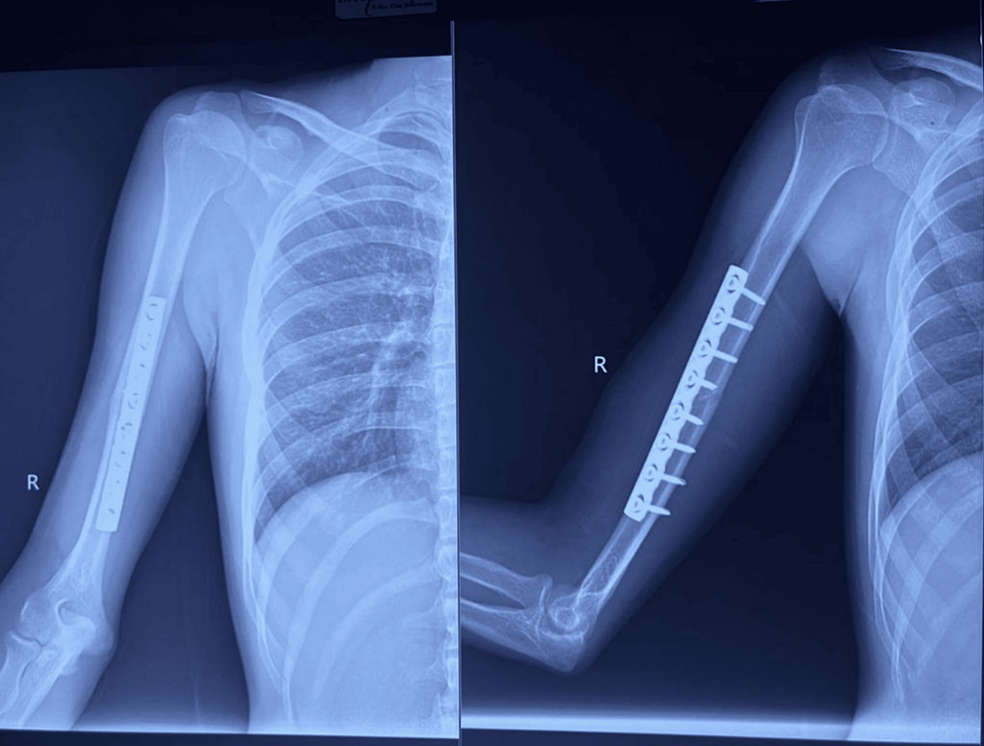
Follow-up radiograph at six months

**Figure 7:**
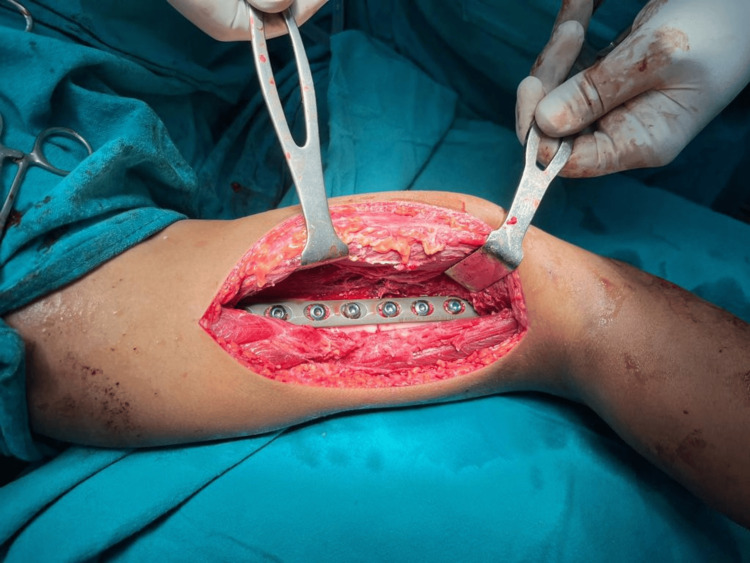
Intra-op image showing placement of LCDCP on the anteromedial surface LCDCP: limited contact dynamic compression plates

After the primary stabilization of patients, thorough history taking and examination were done. A thorough radiological and clinical evaluation was done and patients were allotted the group according to a random number table already generated. Fractures were classified according to the Arbeitsgemeinschaft für Osteosynthesefragen/Orthopedic Trauma Association (AO/OTA) classification system. After the pre-anaesthetic checkup, written and informed consent was taken and patients were taken to the operation theatre. All patients received a prophylactic dose of 1gm ceftriaxone intravenously before induction of anaesthesia. The entire limb is prepared with betadine and spirit, and draped, with both shoulder and elbow exposed. For anterolateral plating (Figures 1-4), the humerus was approached through standard Henry’s approach. The incision was made on the lateral border of the biceps with sufficient length to allow insertion of the plate. The fascia over the biceps muscle was split open. The underlying brachialis muscle was split longitudinally to the bone, and retracted subperiosteally, the lateral half to the lateral side and medial half to the medial side, while flexing the elbow to the right angle. Reduction of the fracture and plate fixation on the anterolateral surface. A 4.5 mm or 3.5mm LCDCP or LCP were used. Hemostasis was achieved and closure of the wound with or without a drain was done. An immediate postoperative radiograph was taken. For anteromedial plating (Figures 5-7), the difference in technique was that the fascia over the biceps muscle was split open and the biceps retracted medially. The underlying brachialis muscle was elevated from its medial margin, lifting along with the musculocutaneous nerve. The radial nerve can be located either at the lateral edge of the brachialis muscle or inside the lateral part of the muscle. The arm was externally rotated to facilitate the visualization of the anteromedial surface of the humerus. The post-operative protocol was the same for both groups with mobilization of the shoulder and elbow on the very next day. Patients were followed up in OPD and assessed on the 14th day, six weeks, three months, and six months using Rodriguez-Merchan (RM) criteria and radiographs.

## Results

In the present study, 23 cases were treated with anterolateral plating and 23 were treated with anteromedial plating the majority of the studied cases were in the age group less than 30 years with mean age 35.7±14.8 and 33.6±11.9 years respectively for anterolateral and anteromedial group. There was male predominance in both the groups but the difference was statistically insignificant (p>0.05). The distribution of the studied cases was done based on AO/OTA classification (Table 1) and it was found that the majority of the cases were in the 12A3 classification i.e., 60.9% (n=14) in anterolateral and 34.8% (n=8) in the anteromedial plating group followed by 12A2 and 12A1 but the difference was statistically insignificant (p>0.05). Our findings were consistent with the findings of Manual S et al. [1], who reported that the most common fracture pattern noted in their study was A3 type followed by A2 type according to AO/OTA classification.

**Table 1:** Distribution of the studied cases based on AO/OTA classification p<0.05 is considered significant. AO/OTA: Arbeitsgemeinschaft für Osteosynthesefragen/Orthopedic Trauma Association

Variables		Anterolateral (n=23)	Anteromedial (n=23)	p-value
AO/OTA Classification	12A1	3 (13.0)	5 (21.7)	0.399
	12A2	4 (17.4)	5 (21.7)	
	12A3	14 (60.9)	8 (34.8)	
	12B1	1 (4.3)	4 (17.4)	
	12B2	1 (4.3)	1 (4.3)	

The mean duration of surgery for anterolateral plating was 55.8±6.4 minutes whereas for anteromedial plating it was 52.1±5.6 minutes (Table 2) which was significantly lower than anterolateral plating (p=0.043). Also, the mean blood loss (Table 2) was significantly higher in anterolateral plating (200.1±10.9ml) than in anteromedial plating (193.6±6.5ml). Kumar ABS et al. [8] supported our findings and reported that the mean duration of surgery for anterolateral humerus plating was 74.2± 9.31 minutes and that for anteromedial humerus plating was 55.5± 5.00 minutes (p<0.01). According to Shahid MZI et al., the mean duration of surgery in Group A (anterolateral surface) was 60.25±7.56, and in Group B (anteromedial surface) was 52.34±10.29. (p<0.001) [3].

**Table 2:** Comparison of mean duration of surgery and mean blood loss among the groups p<0.05 is considered significant. The mean duration of surgery was 55.8±6.4 mins in the anterolateral plating group as compared to 52.1±5.6 mins in the anteromedial plating group and the difference was statistically significant (p=.043). The mean blood loss was 200.1±10.9 ml in the anterolateral plating group as compared to 193.6±6.5 ml in the anteromedial plating group and the difference was statistically significant (p=0.018).

Variables	Anterolateral (n=23)	Anteromedial (n=23)	p-value
The mean duration of surgery (min)	55.8±6.4	52.1±5.6	0.043
Mean blood loss (ml)	200.1±10.9	193.6±6.5	0.018

Rai SK et al. reported that fracture exposure time and amount of blood loss are significantly less in the application of a plate on a flat medial surface as compared to application on the anterolateral surface (p<0.05) [2].

There was no case of non-union and radial nerve palsy in anteromedial plating cases whereas in anterolateral cases one patient did not achieve union and two (8.7%) had radial nerve injury, which recovered completely by the end of the study (Table 3). The union was achieved in the majority of the cases of the anteromedial plating group within 12 weeks (78.3%, n=18) with a mean union time of 11.7±1.5 weeks than the anterolateral group (56.5%, n=13) with a mean union time of 12.3±1.8 weeks. Pain with activity was sensed in 17.4% (n=4) of cases of anterolateral plating than in 8.7% (n=2) cases of anteromedial plating (Table 3). The difference was found statistically insignificant (p>0.05). According to Kumar ABS et al., iatrogenic radial nerve lesions happened in five out of 47 patients (10.6%) whose plates were positioned on the anterolateral humeral surface, but anteromedial plating, which was done on 39 patients, did not cause any radial nerve palsies. Anterolateral humeral plating patients had a non-union rate of 4.3%, which was nearly identical to anteromedial humeral plating patients' 5.1% non-union rate, which was likewise not statistically significant [8].

**Table 3:** Distribution of the studied cases based on the union achieved p<0.05 is considered significant. In the anterolateral plating group, the mean time to reach union was 12.3±1.8 weeks as compared to 11.7±1.5 weeks in the anteromedial plating group (p>0.05).

Variables		Anterolateral (n=23)	Anteromedial (n=23)	p-value
Complications	Non-union	1 (4.3)	0 (0.0)	0.201
	Radial Nerve Injury	2 (8.7)	0 (0.0)	
	None	20 (87.0)	23 (100.0)	
Union in weeks	10	6 (26.1)	8 (34.8)	0.521
	11	1 (4.3)	1 (4.3)	
	12	6 (26.1)	9 (39.1)	
	14	8 (34.8)	5 (21.7)	
	15	1 (4.3)	0 (0.0)	
Mean Union in Weeks		12.3±1.8	11.7±1.5	0.216
Pain	None	16 (69.6)	15 (65.2)	0.427
	Occasional	3 (13.0)	6 (26.1)	
	With Activity	4 (17.4)	2 (8.7)	

A study conducted by Kirin I et al., comparing anteromedial and anterolateral plating of the humerus, also reports no post-operative radial nerve palsy in the anteromedial plating [9].

The patients were assessed based on elbow range movement and shoulder range movement and it was found that the anteromedial plating showed better movement as compared to anterolateral plating but the difference was found statistically insignificant (p>0.05). No disability was found in 69.6% (n=16) and 65.2% (n=15) cases respectively for the anterolateral and anteromedial groups, minimum disability was in 13% (n=3) and moderate in 17.4% (n=4) in the anterolateral group whereas in 21.7% (n=5) and 13.0% (n=3) in the anteromedial group (p>0.05). Shahid MZI et al. [3] reported that six weeks after the surgery we also measured the shoulder and elbow range of motion in all patients. In Group A (anterolateral) there was a flexion deficit of 10 degrees in three patients while normal movements were achieved in all patients in Group B (anteromedial).

On the basis of functional assessment according to RM criteria, the excellent outcome was achieved in 69.6% (n=16) and 65.2%(n=15) in the anterolateral and anteromedial plating groups, respectively. Around 13.0% (n=3) and 21.7% (n=5) show good outcomes whereas, 17.4% (n=4) and 13.0% (n=3) show fair outcomes, respectively in the anterolateral and anteromedial plating groups with insignificant differences (p>0.05) (Table 4). Our findings were comparable to the findings of Prabin N et al., who reported that the RM criteria showed a majority of patients had excellent, n=23 (52.3%) and good, n=15 (34.1%) functional outcomes [10].

**Table 4:** Distribution of cases based on functional assessment (RM criteria). p<0.05 is considered significant. RM criteria: Rodriguez-Merchan criteria

Functional Assessment	Anterolateral (n=23)	Anteromedial (n=23)	p-value
Fair	4 (17.4)	3 (13.0)	0.713
Good	3 (13.0)	5 (21.7)	
Excellent	16 (69.6)	15 (65.2)	

According to Mansoor A et al., the functional outcome was assessed using RM criteria. The excellent functional outcome was achieved in 165 (73.3%) cases and good functional outcome in 58 (25.8%) cases. A fair outcome was achieved in two (0.9%) of the cases [11].

## Discussion

It is recommended to treat the majority of middle-third humerus fractures cautiously, with good union rates and satisfactory functional outcomes, plate osteosynthesis is still the gold standard for fixing humeral shaft fractures.

Even though there are several primary and secondary reasons for surgical fixation as well as several ways to fix the fracture, Henry describes two surgical methods utilized for the humerus: the posterior approach and the anterolateral approach. When treating middle third and proximal third fractures, the anterolateral technique works well. For distal third fractures, the posterior approach is the most appropriate. One can use an anterolateral technique to reach the humerus shaft in its entirety. The radial nerve may sustain injury if the plate is positioned on the humerus' anterolateral surface [3].

The comparatively smooth anteromedial surface allows for robust plate fixation using the anterolateral approach with good union rates and little difficulty without pre-bending the plate [7].

In our study, 23 cases were treated with anterolateral plating and 23 were treated with anteromedial plating the majority of the studied cases were in the age group less than 30 years. There was male predominance in both groups. The majority of the cases were in the 12A3 classification (AO/OTA) i.e., 60.9% (n=14) in the anterolateral and 34.8% (n=8) in the anteromedial plating group followed by 12A2 and 12A1. The mean duration of surgery for anterolateral plating was 55.8±6.4 minutes whereas for anteromedial plating it was 52.1±5.6 minutes which was significantly lower than anterolateral plating (p=0.043). Also, the mean blood loss was significantly higher in anterolateral plating (200.1±10.9ml) than in anteromedial plating (193.6±6.5ml). There was no case of non-union and radial nerve palsy in anteromedial plating cases whereas in anterolateral cases one patient did not achieve union and two (8.7%) had radial nerve injury, which recovered completely by the end of the study. The union was achieved in the majority of the cases of the anteromedial plating group within 12 weeks (78.3%, n=18) with a mean union time of 11.7±1.5 weeks than the anterolateral group (56.5%, n=13) with a mean union time of 12.3±1.8 weeks. Pain with activity was sensed by 17.4% (n=4) of cases of anterolateral than 8.7% (n=2) cases of anteromedial cases. In our study, the patients were assessed based on elbow range movement and shoulder range movement and it was found that the anteromedial plating showed better movement as compared to anterolateral plating. On the basis of functional assessment according to RM criteria, the excellent outcome was achieved in 69.6% (n=16) and 65.2% (n=15) in the anterolateral and anteromedial plating groups, respectively. Around 13.0% (n=3) and 21.7% (n=5) show good outcomes whereas, 17.4% (n=4) and 13.0% (n=3) show fair outcomes, respectively in the anterolateral and anteromedial plating groups.

Even though this study shows that with medial plating, there is minimal or no risk of radial nerve injury. The number of patients in our study was small. It requires a large number of patients with a longer duration for assessment.

## Conclusions

Most humerus shaft fractures are now treated surgically, and open reduction and internal fixation either with a plate or intramedullary nail are the conventional treatments. An anterolateral approach with anteromedial surface plating on the flat medial aspect of the humerus is a good technique for fixing humeral fractures. There is a lower chance of radial nerve injury. In addition, compared to anterolateral plating, medial plating results in reduced blood loss and reduced surgical times.
